# Characterization of Changes in Gene Expression and Biochemical Pathways at Low Levels of Benzene Exposure

**DOI:** 10.1371/journal.pone.0091828

**Published:** 2014-05-01

**Authors:** Reuben Thomas, Alan E. Hubbard, Cliona M. McHale, Luoping Zhang, Stephen M. Rappaport, Qing Lan, Nathaniel Rothman, Roel Vermeulen, Kathryn Z. Guyton, Jennifer Jinot, Babasaheb R. Sonawane, Martyn T. Smith

**Affiliations:** 1 Superfund Research Program, School of Public Health, University of California, Berkeley, California, United States of America; 2 Division of Cancer Epidemiology and Genetics, National Cancer Institute, National Institutes of Health, Bethesda, Maryland, United States of America; 3 National Center for Environmental Assessment, Office of Research and Development, US EPA, Washington, DC, United States of America; 4 Institute of Risk assessment Sciences, Utrecht University, Utrecht, The Netherlands; National Institute of Environmental and Health Sciences, United States of America

## Abstract

Benzene, a ubiquitous environmental pollutant, causes acute myeloid leukemia (AML). Recently, through transcriptome profiling of peripheral blood mononuclear cells (PBMC), we reported dose-dependent effects of benzene exposure on gene expression and biochemical pathways in 83 workers exposed across four airborne concentration ranges (from <1 ppm to >10 ppm) compared with 42 subjects with non-workplace ambient exposure levels. Here, we further characterize these dose-dependent effects with continuous benzene exposure in all 125 study subjects. We estimated air benzene exposure levels in the 42 environmentally-exposed subjects from their unmetabolized urinary benzene levels. We used a novel non-parametric, data-adaptive model selection method to estimate the change with dose in the expression of each gene. We describe non-parametric approaches to model pathway responses and used these to estimate the dose responses of the AML pathway and 4 other pathways of interest. The response patterns of majority of genes as captured by mean estimates of the first and second principal components of the dose-response for the five pathways and the profiles of 6 AML pathway response-representative genes (identified by clustering) exhibited similar apparent supra-linear responses. Responses at or below 0.1 ppm benzene were observed for altered expression of AML pathway genes and *CYP2E1*. Together, these data show that benzene alters disease-relevant pathways and genes in a dose-dependent manner, with effects apparent at doses as low as 100 ppb in air. Studies with extensive exposure assessment of subjects exposed in the low-dose range between 10 ppb and 1 ppm are needed to confirm these findings.

## Introduction

Benzene is a component of gasoline, and the starting ingredient in the production of plastics and polymers via styrene; of resins and adhesives via phenol; and, in the manufacture of nylon via cyclohexane. It is toxic to the bone marrow and is associated with various hematological cancers [1], [2].

Multiple possible mechanisms of action are thought to be involved in benzene toxicity [3], [4], [5], [6]. Benzene exposure has been shown to cause hematotoxicity [7], induce formation of protein adducts [8], [9], and increase the risk of leukemia [10], in a dose-dependent manner. Linear or supra-linear dose-dependent effects on lymphocyte counts and colony formation from myeloid stem and progenitor cells and gene expression were reported at relatively low levels of occupational exposure (≤1 ppm to >10 ppm) in exposed human populations [7], [11], [12]. Recently, through transcriptome profiling of peripheral blood mononuclear cells (PBMC), we reported dose-dependent effects of benzene on gene expression and biochemical pathways in 83 workers exposed to air benzene levels across four concentration ranges (from <1 ppm to >10 ppm), compared with 42 subjects not occupationally exposed to benzene [13]. A 16-gene signature associated with all levels of benzene exposure exhibited an apparently supra-linear dose response. In addition, several immune response-related pathways and the pathway associated with AML were significantly modulated across several of the benzene dose ranges examined.

A deeper understanding of the dose-dependent, disease-relevant human biochemical responses resulting from benzene exposure, particularly at low doses, is important for next generation approaches to human health risk assessment. Therefore, the goal of the current study was to further characterize the dose-dependency of low-dose effects of benzene on genes and biochemical pathways identified in our recent benzene-related microarray analyses of PBMC [13]. Specifically, continuous data for individual benzene exposure across all dose groups was used to generate dose-response curves on a continuous scale. We used predicted measures of benzene exposure in the group of subjects with only ambient exposure to benzene. These subjects were regarded as controls in the previous study but were, in fact, non-occupationally exposed to benzene at varying, relatively low environmental concentrations. Inclusion of data from these individuals allowed us to examine more closely the responses in the low dose (environmental) region of exposure. Using data from all 125 study subjects, we applied non-parametric approaches, based on the SuperLearner [14], to fit the responses of individual gene expression as a function of benzene exposure. The use of non-parametric approaches is particularly relevant here and in epidemiological studies in general because it is impossible to know the exact functional relationships among the variables such as gene expression, dose from exposure, age, gender and smoking status of the subject, cell counts etc. Non-parametric approaches make minimal assumptions about these functional relationships and let the observed data guide the choice of the best models using rigorous statistical criteria (*e.g*., cross-validation [14]). The implication of making parametric assumptions is that if these assumptions are untrue (which is almost certainly the case), the results produced can be difficult to interpret. In the current study, we developed novel non-parametric approaches to model the responses in biochemical pathways of interest. We chose to model the responses in 5 pathways, including the AML pathway and two other pathways previously shown to be modified by benzene, and two pathways presumably unrelated to benzene exposure. We also employed the models to examine dose-response relationships in the expression of a set of candidate genes known to be associated with AML and with the metabolism of benzene.

The overall goals of this study were to estimate the benzene exposure-response patterns of relevant gene expression and biochemical pathways in a statistically rigorous, non-parametric manner. This approach allowed us to identify consistencies in the shapes of the resulting exposure-response curves and characterize responses particularly in the low-dose region of exposure. Since our original microarray data were generated from PBMCs which comprise various cell types [15], including T lymphocytes (CD4 and CD8 ∼65%), B cells (∼15%), natural Killer cells (∼10%), and monocytes (∼10%), we adjusted for changes in percentages of these subtypes after benzene exposure in our analyses.

## Materials and Methods

**Figure 1:**
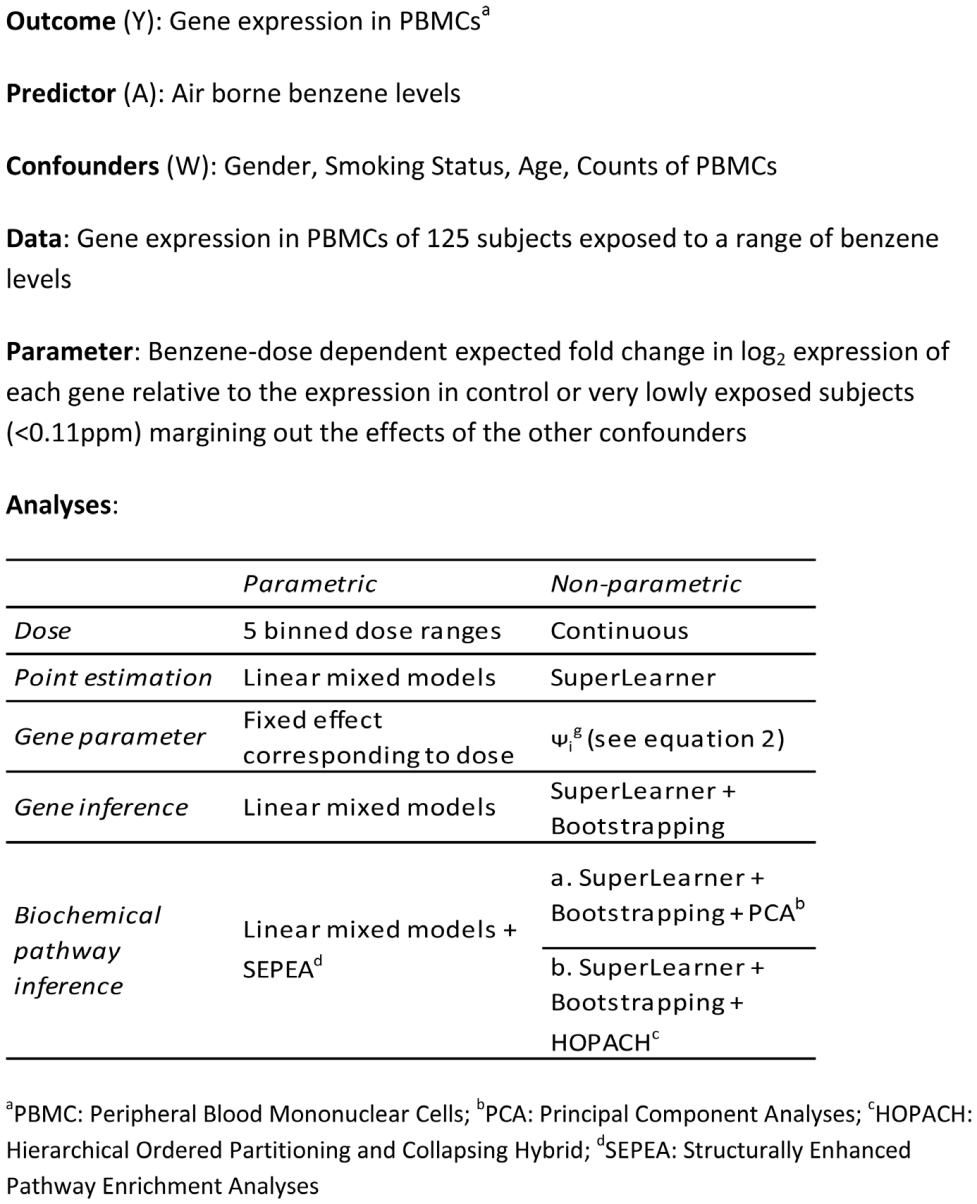
Overview of methods and analyses.

A brief overview of the data and methods used is given in Figure 1.

### Data Sets

#### Ethics statement

This study complied with all applicable requirements of U.S. and Chinese regulations, including institutional review board approval at the National Cancer Institute, Bethesda, Maryland USA and the National Institute of Occupational Health and Poison Control, China CDC, Beijing. Participation was voluntary, and written informed consent was obtained.

#### Study population, hematotoxicity, and gene expression data

The overall molecular epidemiology studies investigating occupational exposure to benzene [7], [16] and the gene expression data [13] upon which the analyses in the current study are based were previously described. The gene expression data were generated through transcriptome analysis by microarray of 125 subjects exposed to various levels of benzene. Among the 125 subjects, 42 were exposed to levels that were below the limit of detection of the benzene monitors (0.04 ppm) used; 29 were exposed to <<1 ppm (average <1 ppm and most individual measurements <1 ppm) benzene; 30 were exposed to <1 ppm (average <1 ppm); 11 were exposed to levels between 5 ppm and 10 ppm; and 13 were exposed to levels ≥10 ppm. For each of the exposed individuals in the study, benzene exposure was estimated in terms of the average air-benzene level (in units of parts-per-million). The exposure levels of the 42 subjects that were below the limit of detection were estimated using unmetabolized urinary benzene levels, as previously described [17]. Complete blood cell counts, including counts for CD4 and CD8 T lymphocytes, B lymphocytes, NK cells and monocytes, the major cell subsets of PBMCs, were available for all the individuals analyzed by microarray [7].

### Biochemical Pathways

The biochemical pathways analyzed in this study were obtained from the Kyoto Encyclopedia of Genes and Genomes (KEGG) Pathway database [18], [19], [20]. The data for the set of genes within each pathway and their associated interactions were downloaded using the KEGG application programming interface (http://www.kegg.jp/kegg/soap/). Five pathways were analyzed, including three (AML, B-cell receptor signaling and Toll-like receptor signaling) previously shown to be differentially modulated with benzene exposure [13] and two (Steroid hormone biosynthesis and Maturity onset of diabetes) presumably unrelated to benzene exposure were not differentially modulated.

### Linear Mixed Effect Models

We conducted variance components analysis using a linear mixed model [21] to assess the proportion of total variation due to differences between subjects, hybridizations, labels, and chips, both before and after normalization [quantile normalization in the Affy package [22] in R [23]]. For each probe, we estimated the association between exposure level and expression level using a mixed-effects model with random intercepts that accounted for clustering by subject, hybridization, and label. The fixed effects in our model included gender (1 = male, 0 = female), current smoking status (1 = yes, 0 =  no), age (in years, linear term), B cells, Natural Killer (NK) cells, monocytes, and CD4 and CD8 cells (as counts and included as linear terms). These were potential confounders of associations (denoted by the vector of random variables, *W*) between logarithm to the base 2 of gene expression (denoted by the random variable, *Y*) and benzene exposure in five dose ranges (denoted by the random variable, *A*). The model is thus given by,
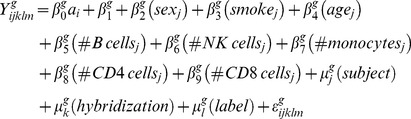
(1)

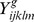
 denotes the log_2_ of the g*^th^* gene expression, at the dose 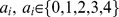 obtained from the *j^th^* subject after the *k^th^* hybridization, *l^th^* labeling step in the microarray sample preparation and the *m^th^* chip. The 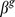 parameters denote the fixed effects associated with the respective covariates; the 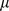 parameters denote the random effects, and 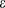 denotes the normally distributed error associated with the model. 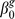, the fixed effect associated with benzene exposure, is the parameter of interest in the model. We fitted this mixed-effects model in R with the lmer function in the *lme4* package [24]. We also fit the mixed effects model without cell counts as potential confounders as given in Equation (2).



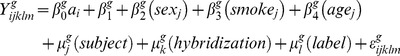
(2)We identified differentially expressed probes as those with a statistically significant log-fold change (based on likelihood ratio tests). We computed *p*-values adjusted for multiple testing by controlling the false discovery rate (FDR) with the Benjamini-Hochberg procedure [25], using the *multtest* package in R. These FDR-adjusted *p*-values ≤0.05, the traditional experiment-wise type I error rate, were considered significant.

### Pathway Enrichment Analysis

We used a method known as “structurally enhanced pathway enrichment analysis” (SEPEA_NT3) [26], which incorporates the associated network information of KEGG (Kyoto Encyclopedia of Genes and Genomes) human biochemical pathways [19], [27], [28]. Unlike traditional pathway enrichment methods that treat pathways as sets of genes, SEPEA treats pathways as networks of interacting proteins and/or enzymes. The genes corresponding to the proteins in the signaling network are given more weight according to whether they are at the receptor or the terminating end of the pathway that typically signals for transcription in a number of genes. Further, pathways where the perturbed genes are close relative to each other on the associated network are modeled as being more likely to be affected than pathways where he perturbed genes occur further apart over the network. The significance obtained by SEPEA_NT3 was based on 10000 randomizations.

### Bootstrap-SuperLearner

The SuperLearner [14] method is a theoretically optimal (relative to the so-called Oracle estimator) approach to model selection in a data adaptive manner. This method requires a set of different statistical learning algorithms that a user could consider as being appropriate models of the data. SuperLearner then uses a cross-validation-based loss function to estimate an optimal combination of predictions from the different input algorithms to produce model fits. The SuperLearner was used to fit 
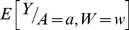
 where Y, A and W have the same meaning as in the previous sections. Note in these analyses cell counts are included as additional confounders. The fits were computed in the SuperLearner package [29] implemented in the R statistical environment [23] with a choice of a 10-fold cross-validation-based loss function. The statistical learning algorithms used were random forests [30], multivariate adaptive regression splines [31],, bagging [32], Bayesian Generalized Linear Models [33], cforests [34], [35], [36], neural networks [37], loess regression [38] and support vector machines [39]. Different parameter settings for each of these algorithms in their respective R packages were used (see Table S1)In total there were 32 learning algorithms. The reader interested in implementing the SuperLearner is referred to a vignette (http://cran.r-project.org/web/packages/SuperLearner/vignettes/SuperLearnerPresent.pdf) describing its implementation in R.

In order to determine the variability of the SuperLearner mean response estimates, a bootstrapping procedure was implemented. Let *n* denote the number of subjects and *N_BS_* denote the number of bootstrap samples. 1000 bootstrap samples were chosen in all cases. Then for each bootstrap sample, *n* subjects are drawn randomly with replacement and the mean response is then estimated using the SuperLearner for each of the bootstrap samples.

The marginal association of a given response (expression of gene *g*) with the *i^th^* dose of benzene exposure (corresponding to *A = a*) was then estimated by,
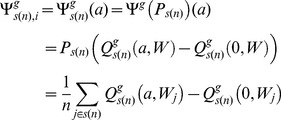
(3)Where 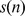 represents a bootstrap sample, 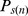 represents the empirical distribution based on that sample, 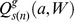 the estimate of 
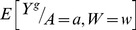
 based on the SuperLearner applied to 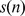. 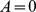 represents doses ≤0.11 ppm. So for every 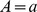
(at which we calculated 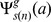, which were at points separated 0.5 units apart on the log_2_ dose range), we get the average difference of the predicted log_2_ gene expression value (averaged across the W) and the predicted log_2_ gene expression value if the sample represented very low to no exposure.

All the subjects with undetectable benzene exposure levels in this study had predicted air benzene exposures less than 0.11 ppm. Under certain assumptions [40], [41], this parameter of interest in Equation (3) also has also a simple causal interpretation, i.e., it represents the mean log fold change of a given gene’s expression due to exposure to a given dose relative to those with exposure less than 0.11 ppm air benzene levels.

### Biochemical Pathway Response

The derivation of the non-parametric estimate of the mean response of a biochemical pathway with an outcome of interest in the presence of confounders to its constituent gene expressions is a statistical problem that does not appear to have been addressed before in the literature. Examples of model-based approaches include those in [42], [43] who proposed generalized linear model-based approaches to test for pathway association with a binary clinical outcome and survival times. We propose two ideas to provide summary responses of the expression of all the genes in a biochemical pathway, both of which use non-parametric estimates from the previously described Bootstrap-SuperLearner approach. The first idea is based on using principal component analysis (PCA) on the estimates from the SuperLearner of the expressions of genes in the pathway. Principal component analysis has been used in the past to model the pathway response [44], [45]. The change in expressions of the genes due to benzene exposure is potentially confounded by other covariates in the study. Hence it will not be correct to perform a direct analysis of the expressions of the genes in the pathway in order to get a summary response. Therefore, PCA is performed on the SuperLearner-based non-parametric estimates of changes in gene expressions due to benzene exposure. The second idea utilizes a clustering analysis of these SuperLearner estimates in order to identify clusters of gene expression responses and medoid genes or particular genes that have responses that are representative of responses in the identified clusters. Clustering analysis of gene expression data [46] has been more or less standard for more than ten years.

Assume that the human biochemical pathways are identified by indices in the set 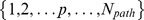 Let *N_p_* denote the number of probes corresponding to genes involved in the given pathway identified by the index *p*. *N_d_* is the number of points on the dose range of the exposed individuals where the SuperLearner [14] estimates are computed. Equally spaced points were chosen, 0.5 units apart on the logarithmic dose range.

In the first analysis, the dose-dependent responses were estimated by the first and second principal components (or first and second columns of the 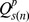 eigenvector matrix) of the covariance matrix, 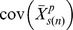 created from the N_p_×N_d_ matrix, 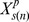 obtained for the SuperLearner [14] estimate of the parameters of interest (see Equations (4)-(6), where 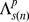 represents diagonal matrix with the corresponding eigenvalues ordered in a decreasing manner). This was done for each bootstrap sample, *s*. Note,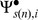 denotes the mean parameter of interest across all the probes at the i^th^ dose level.
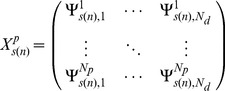
(4)




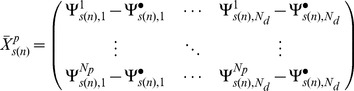
(5)




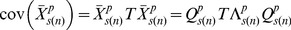
(6)Where
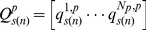
(7)And 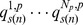 are the *N_p_* eigenvectors corresponding to the eigenvalues in the matrix 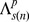. Each of the *N_d_* elements of the first and second eigenvector was taken to represent the pathway response at the corresponding dose. In order to make comparisons of these eigenvectors across all bootstrap samples, two modifications were made to these eigenvectors. First, the first element of the (first or second) eigenvector for each of the bootstrap samples was normalized to zero. Second, if the sign of this normalized eigenvector responses at 0.1 ppm was negative, and then the negative of the elements of normalized eigenvector was plotted. This can be done because any scalar multiple of the reported eigenvector is an equally valid eigenvector for the given eigenvalue. We denote these modified eigenvectors by 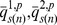 where for *i = 1,2*,

 = 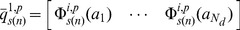
(8)The *p^th^* pathway response at dose *a_j_* is given by 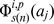. Note by definition 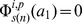.

For each bootstrap sample, these modified normalized eigenvector-based pathway response with dose is plotted as a smoothed cubic spline using a smoothing parameter of 0.5. The plots were made in the R statistical environment [23].

In the second analysis, the dose-dependent responses were presented as a clustered (*N_p_×N_p_*) distance matrix between the probes associated with the genes involved in the pathway. The clustering was performed using the HOPACH algorithm [47] in the package *hopach* [48] in the R statistical environment [23] where the distance between the two *N_d_×1* vectors, 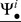 and 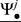 associated with a pair of probes, *i* and *j* is measured by the cosine distance metric. Using this distance metric, HOPACH builds a hierarchical cluster of trees by recursively partioning the data set. Note the analyses here were performed on the matrix 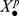 that represents the average of 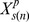 over all the bootstrap samples.
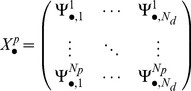
(9)From the clustering analyses, we also presented the responses of the genes identified as medoids for the six largest clusters as identified by the HOPACH algorithm [47].

The advantage of the principal component analyses of the pathway response is a one-picture summary response of the variability of the estimates of mean response of the pattern among a significant proportion of the genes in the pathway. However, this picture does not inform whether the genes are being over or under expressed at different levels of exposure. The plots of the medoid genes provide the sign of response of these chosen genes.

### Estimates of the Change in the Rate of Change of Response

Characteristics of the shapes of the dose-response curves were obtained in terms of the change in the absolute rate of change of marginal effect of benzene exposure from below 1 ppm to above this level. For the two pathway responses (*i = 1,2*), the rate of change of the marginal effect response below 1 ppm for the bootstrap sample *s(n)* is estimated by,
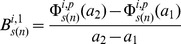
(10)Where 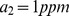 and 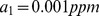, 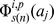 is as given in Equation (8). The rate of change of response above 1 ppm by,
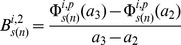
(11)Where 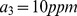 and the estimate of the change in the absolute rate of change of response,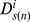 is chosen to be
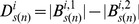
(12)Analogously the estimates of the change in the absolute rate of change of gene, *g* level responses, 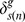 are given by,
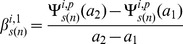
(13)

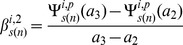
(14)

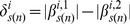
(15)Where 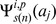 is given by Equation (3). The bootstrap samples of 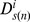 and 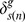 are used to estimate the 95% confidence intervals for *D^i^* and *δ^g^*. Significant positive values of these estimates suggest supralinearity of the marginal effects of benzene exposure while significant negative values suggests sublinearity of these effects between 0.001 ppm and 10 ppm benzene levels.

## Results

### Predicted Air Benzene Exposure Levels in Non-occupationally Exposed Subjects

The air benzene exposure levels for the 42 control subjects [13] were predicted from their urinary unmetabolized benzene levels [17]. For 8 of the control subjects, exposure predictions were unavailable and these subjects were excluded from the non-parametric analyses. The predicted benzene exposure levels ranged from 1.4×10^−4^ ppm to 0.11 ppm, with 32 subjects predicted to have levels below 0.009 ppm.

### Peripheral Blood Cell Counts as Potential Confounders of Gene Expression

**Figure 2:**
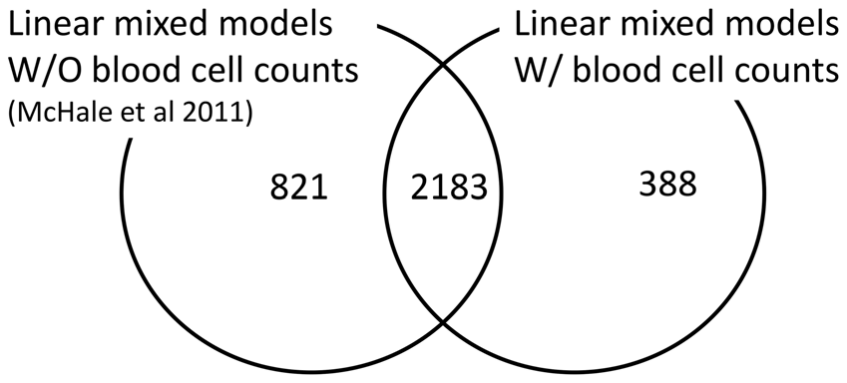
Overlapping sets of genes determined by two linear models. Two linear mixed models were used, a published model [13] (see Equation (2)) and a modified version including counts of different blood cell types as potential confounders of gene expression (see Equation (1)). Differential expression was determined based on altered fold changes in at least one of the four previously chosen dose ranges of benzene exposure, with an FDR-adjusted *p*-value<0.05.

Two linear mixed models of gene expression as a function of air benzene exposure were fitted to the data from 125 subjects who had been exposed to benzene in four previously chosen concentration ranges, <<1 ppm, <1 ppm, >5 ppm and <10 ppm, and ≥10 ppm,or were controls. One of the models was identical to that recently reported by us [13] and included the gender, age and smoking status of the subjects as potential confounders of gene expression. The second model was the same but also included the measured counts of different cell types present in the PBMC as additional confounders (see Equation (1)). The estimates of the fixed effects of this model are given in Table S2. The distribution of the estimates of the intra-class coefficients for each of the random effects do not change when moves from a model that does not include the PBMCs to one that does (see Figure S1). As shown in the Venn diagram in Figure 2, there was a significant overlap (2183 genes) between the sets of genes identified as differentially expressed (FDR-adjusted *p*-value <0.05) by each linear model. Fisher’s exact test estimated a *p*-value <2.2×10^−16^ for the null hypothesis stating the independence between genes declared differentially expressed by the two linear models. When cell counts were incorporated as potential confounders, 821 out of 3004 genes identified as differentially expressed in the original model were no longer significant, while an additional 388 genes were found to be significant. There were also no major differences in the pathway enrichment values for all the KEGG human pathways using results from either model (Table S3). Pathway enrichment analyses were also done on the sets of genes that were commonly identified as differentially expressed genes (2183 genes) and uniquely by either of the models (821 or 388 genes) (see Table S3). The pearson correlation between the log_10_ transformed p-values across pathways for the set of genes uniquely identified by the model that did not incorporate cell count with the corresponding p-values for the set of genes commonly identified by both models is 0.06 (p-value = 0.32). The pearson correlation for the pathway p-values using the set of genes uniquely identified by the model that incorporated cell count with corresponding p-values for commonly identified set of genes is 0.22 (p-value = 0.0005).

### Biochemical Pathway-based Responses

A dose-specific parameter of interest (defined in Equation (3)) was estimated non-parametrically for the expression of each probe/gene in the KEGG AML, B-cell receptor signaling, Toll-like receptor signaling, Steroid hormone biosynthesis, and Maturity onset of diabetes pathways, at points equally spaced 0.5 units apart on the logarithmic dose range. For a given gene at a chosen dose, this parameter is the expected log fold-change in the expression of the gene at that dose relative to the mean expression of subjects exposed to levels below 0.11 ppm. The parameters were estimated using the SuperLearner [14] that used 32 learning algorithms and the sampling distribution of these parameters was estimated via a bootstrapping procedure in which the parameters are re-estimated using random selection (with replacement) of the 125 subjects. The bootstrapping procedure was repeated 1000 times.

**Figure 3:**
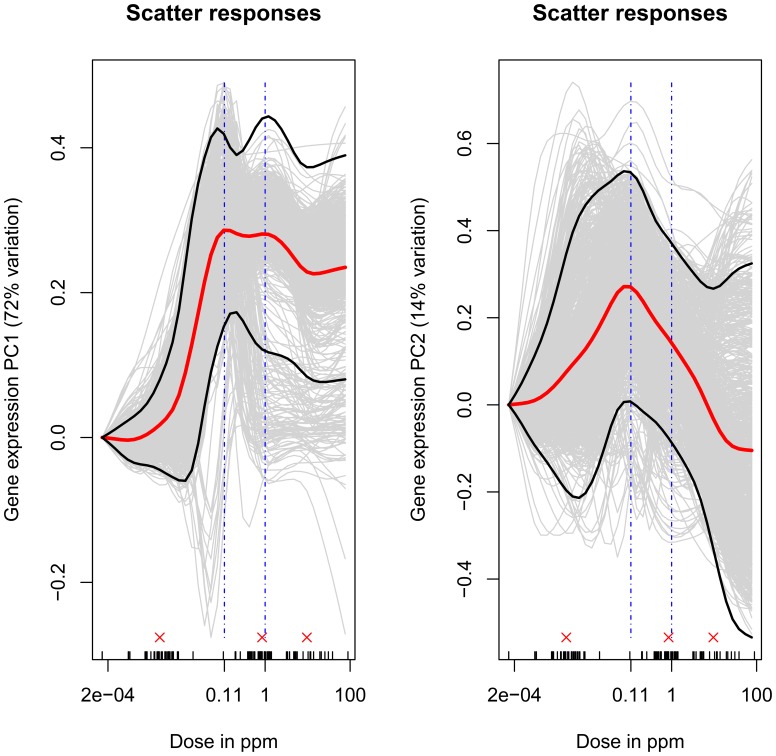
AML pathway-Principal Components-based response. The continuous fits in the two subplots use the first and second eigenvectors (that are slightly modified, see Material and Methods section) respectively from the eigenvector matrix, 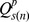 given in Equation (5). The elements of the individual eigenvectors are treated as the pathway response at the corresponding dose. The subscript *p* corresponds to the pathway under consideration and superscript *s* to a given bootstrap sample. The bold red line correspond the mean parameter estimates across the bootstrap samples and the bold black lines represent the corresponding 95% confidence intervals for the mean parameter estimates. The small vertical ticks on the x-axis denote doses to which one or more subjects in the study were exposed and consequently the doses for which data for all covariates under consideration were available. The three red ‘x’s above these ticks indicate the doses that there used to compare the rate of change of the marginal effect of benzene exposure from 0.001 to 1 ppm air benzene to the corresponding rate from 1 to 10 ppm air benzene.

**Figure 4:**
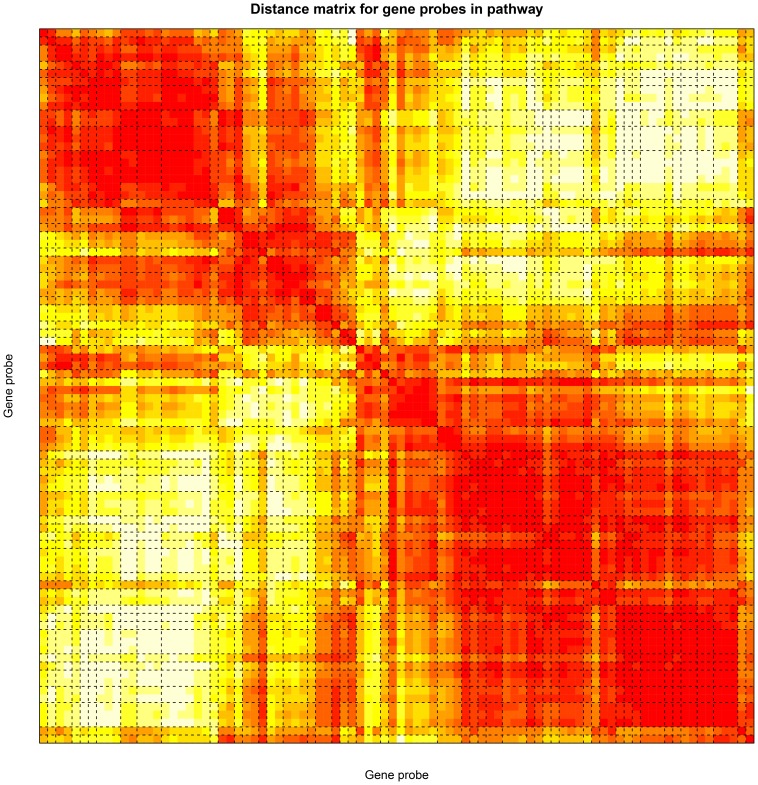
AML pathway-Clusters of probes/genes. Hierarchical cluster of the probes in the AML pathway. The probes are clustered based on the distance between the corresponding rows of the matrix, 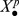 given in Equation (6). The figure is a visual representation of the distance matrix between all the probes/genes in the pathway. The color of the *(i,j)^th^* position of the distance matrix is a measure of how close probes *i* and *j* are to each other based on their response across the dose range. The color ranges from white to red. The closer the pair of probes is two each other, the greater the intensity of red at the corresponding position. The dashed black lines correspond to boundaries of clusters of probes as determined by the HOPACH algorithm [47].

The first and second principal components of the estimated parameters for all genes in the AML pathway, evaluated across the entire study dose range, are shown in Figure 3. Together, these two principal components captured 86% of the dose-dependent variation in expression of genes in the AML pathway. The first and second principal components of the dose-response parameters for all genes in the B-cell receptor signaling, Toll-like receptor signaling, Steroid hormone biosynthesis and Maturity onset of diabetes pathways are shown in figure S2, respectively. Visually, the mean estimates of the first principal components of the responses look similar across the five chosen pathways and suggest supra-linear responses. This is quantitatively reinforced by the fact that the estimates of the change in the absolute rate of change of marginal effect of benzene exposure from below 1 ppm to above 1 ppm for the first principal component of each of the five pathways (see Equation (12)) are significantly positive (p-value <0.05) (see Table 1 and Table S4). There are suggestions of responses at doses below 0.1 ppm, and exposures of around 0.1 ppm and 1 ppm appear to be inflection points for at least three (AML, Toll-like receptor signaling and Maturity Onset of Diabetes) of the five pathways. The mean estimates of the second principal components are also similar across the five pathways, with exposures around 0.1 ppm appearing to represent inflection points for these responses. The responses of the probes in the pathway were clustered using HOPACH [49] (Figure 4 and figure S3), and the results confirm that many sets of genes exhibit similar response patterns.

**Table 1:** Median and 95% confidence interval (CI) estimates of the rate of change of marginal effect of benzene exposure below 1 ppm (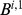/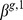 – see equations (9) and (12)) and above 1 ppm (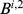/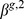 - see equations (10) and (13)) and the change in absolute rate of change of the marginal effects from below 1 ppm to above 1 ppm (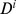/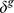 – see equations (11) and (14)) for the first two principal components of the Acute Myeloid Leukemia pathway and six chosen genes of interest.

	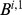 /β^g,1^		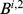 /β^g,2^		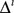 /δ^g^	
Pathway/Gene	Median	95% CI	Median	95% CI	Median	95% CI
Acute Myeloid Leukemia: Principal Component 1	0.333	(0.008, 0.394)	−0.006	(−0.016, 0.002)	0.328	(0.019, 0.380)
Acute Myeloid Leukemia: Principal Component 2	0.106	(−0.244,0.349)	−0.024	(−0.040,0.010)	0.108	(−0.012,0.353)
RUNX1	0.07	(0.018, 0.158)	0.004	(−0.008, 0.025)	0.063	(0.010, 0.151)
FLT3	−0.079	(−0.204, −0.012)	0	(−0.005, 0.004)	0.078	(0.011, 0.202)
CEBPA	−0.877	(−1.11, −0.584)	0.03	(0.006, 0.069)	0.847	(0.561, 1.068)
LEF1	0.032	(−0.035, 0.120)	−0.009	(−0.019, −0.002)	0.025	(−0.008, 0.106)
CYP2E1	0.051	(−0.004, 0.147)	−0.002	(−0.007, 0.002)	0.049	(0.002, 0.146)
CYP2F1	0.002	(−0.033, 0.03)	−0.001	(−0.004, 0.001)	0.008	(−0.001, 0.038)

### Expression-based Responses of Chosen Genes of Interest

**Figure 5:**
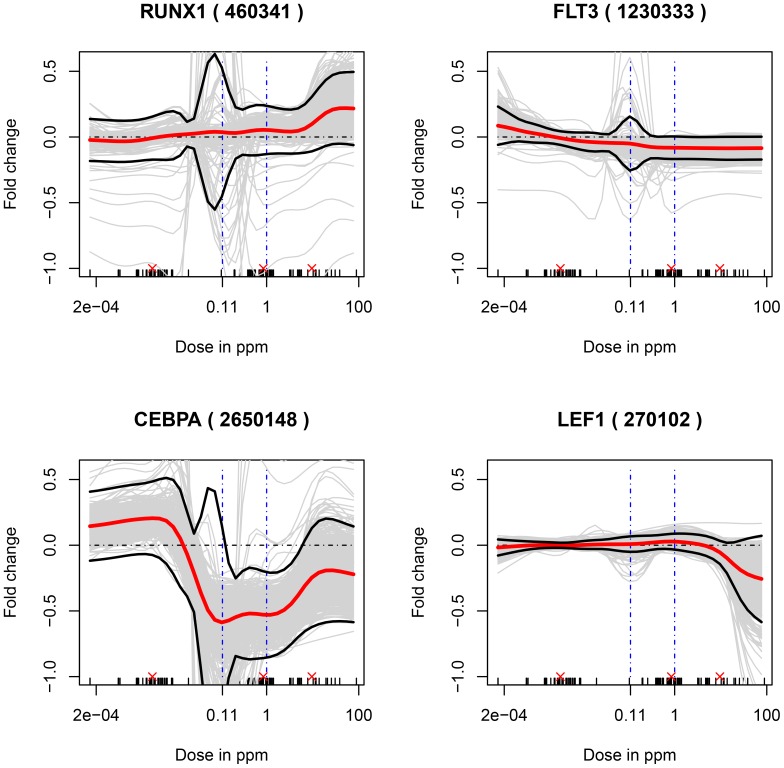
Responses of selected genes associated with the leukemia disease process. Non-parametric model fits to the expression response of the probes corresponding to six genes known to be associated with AML, with air-benzene concentrations in parts per million. Note the responses here are log fold-changes in expression. The dot-dashed horizontal line at a log fold change value equal to zero indicates the no-effect response. The gene names along with the corresponding probe id number on the microarray in parentheses are provided for each gene. The small vertical ticks on the x-axis denote doses to which one or more subjects in the study were exposed and consequently the doses for which data for all covariates under consideration were available. The three red ‘x’s above these ticks indicate the doses that there used to compare the rate of change of the marginal effect of benzene exposure from 0.001 to 1 ppm air benzene to the corresponding rate from 1 to 10 ppm air benzene.

**Figure 6:**
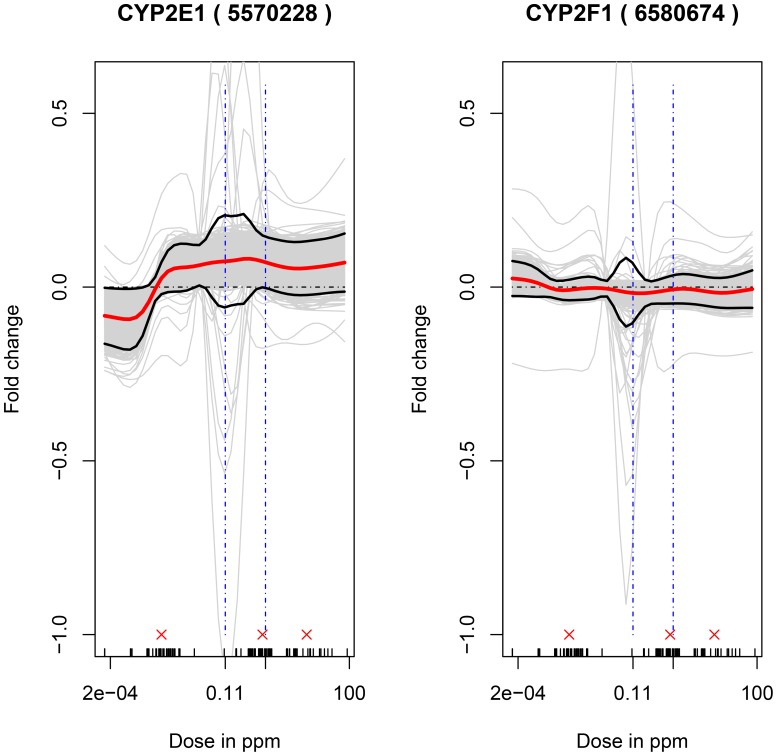
Response of two Cytochrome p450 genes associated with benzene metabolism. Non-parametric model fits to the expression response of the probes corresponding to two genes known to be associated with the metabolism of benzene, CYP2E1 and CYP2F1, with air-benzene concentrations in parts per million. Note the responses here are log fold-changes in expression. The dot-dashed horizontal line at a log fold change value equal of zero indicates the no-effect response. The gene names along with the corresponding probe id number on the microarray in parentheses are provided for each gene. The small vertical ticks on the x-axis denote doses to which one or more subjects in the study were exposed and consequently the doses for which data for all covariates under consideration were available. The three red ‘x’s above these ticks indicate the doses that there used to compare the rate of change of the marginal effect of benzene exposure from 0.001 to 1 ppm air benzene to the corresponding rate from 1 to 10 ppm air benzene.

A small set of genes was chosen based either on their known association with leukemogenesis or because they code for enzymes putatively associated with the metabolism of benzene to its toxic metabolites. In the first class, oncogenes (RUNX1, FLT3, LEF1) or tumor suppressors (RUNX1, CEBPA) implicated in leukemia [50], [51], [52], [53], [54], [55] were selected. RUNX1 has been identified as both an oncogene and a tumor suppressor gene [56], [57]. The responses (in terms of log-fold changes) of these genes are plotted in Figure 5. Among these genes, RUNX1, LEF1 and CEBPA were differentially expressed (FDR<0.05) based on the linear model in Equation (1) across four binned dose ranges. Based on the position of the line corresponding to no change (i.e., log fold change equals zero) relative to the 95% confidence interval of the estimated fold change at a chosen dose, the expressions of FLT3 and CEBPAare all down regulated on exposure to levels of air benzene above around 0.1 ppm. RUNX1, FLT3 and CEBPA display profiles that are supralinear as captured by the significant positive values of the change in the absolute rate of change of marginal effect of benzene exposure from below 1 ppm to above 1 ppm (see Equation (15) and Table 1). The responses of the expression of two genes, CYP2E1 and CYP2F1 (enzymes putatively associated with the metabolism of benzene to its toxic metabolites), are shown in Figure 6. CYP2E1 is known to be involved with the metabolism of benzene [58], [59], [60] and CYP2F1 has been hypothesized to be associated with benzene metabolism [3]. CYP2E1 expression was increased at levels of air benzene concentration as low as 0.01 ppm, while expression of CYP2F1 was largely unaltered.CYP2E1, but not CYP2F1, was found to be differentially expressed (FDR<0.05) based on the linear model in Equation (1) across four binned dose ranges. The dose-response of CYP2E1 is supralinear (see Equation (15) and Table 1).

## Discussion

The analyses in this study sought to further characterize the dose-dependency of changes in gene expression associated with occupational exposure to benzene that we reported recently [13] and to extend them into the ambient environmental range by estimating exposures for the non-occupationally exposed ‘controls’. We further extended the analyses to include PBMC subset cell counts as potential confounders of expression.

Significant overlap was seen for the majority of genes identified as differentially expressed using the parametric linear models, regardless of whether or not PBMC cell counts were incorporated as potential confounders. Genes that did not remain differentially expressed after incorporation of cell counts as confounders could be indirectly related to benzene-induced cell count decrements. One plausible example is the CD44 gene; it encodes a marker for CD4 and CD8 cells, both of which were reduced in number in benzene-exposed individuals [7]. However, the estimation of additional parameters in the model to incorporate cell counts as confounders resulted in loss of statistical power to identify genes as differentially expressed. Conversely, the incorporation of cell counts in the model may improve the model fit and subsequently increase the ability of the model to detect true dose-specific changes in gene expression. This is partly suggested by the significant but relatively small correlation (0.22) of the pathway p-values using the set of genes uniquely identified by the model incorporating cell counts with the pathway p-values using the set of genes commonly identified by both models. In either case, the fact that a significant overlap was observed implies that the majority of the changes in gene expression are not directly mediated through the hematotoxicity of benzene.

We defined the dose-dependent effects on gene expressions as our parameters of interest as a marginal effect of benzene exposure. The definition of the parameter in dose-response studies of this kind is to the best of our knowledge novel in the toxicology literature – that is, as the marginally adjusted curve of the mean outcome versus exposure. This is extremely important in itself, because when measuring how dose-response affects a population, one should estimate a population level dose response parameter. However, what is typically done is to estimate the dose response in a parametric or semi-parametric model, where the resulting curve represents potentially a different curve for every covariate group. To avoid this problem, the predominant approach is to simply make the simplifying, but erroneous assumption, that all groups have the same dose response curve). We wanted to make no such bias inducing assumptions, and so we estimated these dose response curves in a nonparametric model, using optimal data-adaptive methods (SuperLeanrer [14]) for estimating the relationship of expression to exposure and the confounders. Thus, we have approached a problem typically approached using ad hoc methods, based on arbitrary statistical modeling assumptions, and used methods based on what is truly known about the relationship of expression to the covariates including exposure, that is nearly nothing, and derived optimal (the SuperLearner is not ad hoc, but based on the theorem of the Oracle Inequality [14]) estimators respecting the underlying knowledge. We use a bootstrapping approach in order to estimate the sampling variability of these estimates. Note that these estimates are essentially derived from data-adaptive methods in a very large (nearly non-parametric) model, where the number of assumptions made about the probability generating distributions is minimal, particularly as compared to standard approaches using parametric models.

The five pathways chosen for analyses were selected based on the results to our earlier analyses [13] with the same gene expression data on the same set of KEGG [19], [27], [28] human pathways. Ideally, we should have performed these analyses while being agnostic to the potential biochemical pathways being targeted. This would mean analyzing all 22177 probes on the microarray by the proposed non-parametric methodology. However, we chose to analyze the 5 pathways in part because the proposed non-parametric methodology requires significant computing power- one bootstrap sample run of the dose response of a given probe/gene using the SuperLearner [14] that ran 32 learning algorithms took around 20 seconds to run on a 4-core linux machine with around 1 GHz cpu and 16 GB RAM. We should note that the associations of benzene exposure are being made with biochemical pathways of diseases (AML and Maturity Onset of Diabetes) and not with the diseases directly. These biochemical pathways represent a summary of the literature on the specific disease pathogenesis. Further the exact definition of a specific biochemical pathway in terms of its constituents and associated interactions will be consistent though not the same across different pathway databases. Therefore our choices of the pathways were from the same set of pathways analyzed before albeit with the same data – our goal here being a better characterization of the dose responses over the entire continuous range of exposures. The AML pathway was of particular interest because of the established association of benzene exposure with leukemia incidence [1], [2]. The other two pathways (B-cell receptor and Toll-like receptor signaling) were randomly chosen from the set of pathways which displayed significant dose response over the range of benzene exposures. Similarly the Steroid Hormone Biosynthesis and Maturity onset of diabetes pathways were chosen from the list of pathways that did not display statistical significant responses.

The mean estimates of first and second principal components of the dose-response relationships determined by the our method method for all genes in the five chosen pathways (AML, B-cell receptor signaling, Toll-like receptor signaling, Steroid hormone biosynthesis and Maturity onset of diabetes) showed apparent similarities and similar inflection points were observed for several of the pathways. Since two of the analyzed pathways, Steroid hormone biosynthesis and Maturity onset of diabetes, are presumably unrelated to benzene, the noted similarities in response implies that the changes in expression of genes in these pathways are real effects though they were not large enough to provide statistical significance for modulation at the pathway level. Consistency of the shapes of the responses across the five chosen pathways may be a consequence of the correlation among gene expression levels on a system-wide basis through coordinated transcriptional regulation. However, analyses of the binding sites in the promoter regions of the differentially expressed genes (across doses, as determined by the linear model in Equation (1)) did not reveal enrichment of any transcription factor binding sites (data not shown).

Together, these similarities support the plausibility of the observed supra-linear dose-responses. In addition supralinearity is quantitatively supported by positive values of the parameter that captures the change in the absolute rate of change of marginal effect of benzene exposure from below 1 ppm to above 1 ppm (see Equations (12) and (15), Table 1). This adds to the literature of observed supra-linear responses associated with benzene exposure – see for example the response of benzene oxide-albumin adduct formation with benzene exposure [61], the dose related production of benzene metabolites [17] and the relative risk of leukemia with benzene exposure [10].

Our statistical tests for supralinearity are based on comparing the rate of change of the marginal effect of exposure below 1 ppm (0.001–1 ppm) benzene to the rate of change of this marginal effect above 1 ppm (1–10 ppm). We don’t perform statistical tests for supra-linearity of the overall dose response curve. Testing for supra-linearity is a very subtle issue, since any data adaptive approach, compared to some model in a goodness of fit test, will always win out asymptotically (any null model will be not perfectly right, so as sample size grows, and the data-adaptive approach will favor more highly parameterized models to create a better fit, any improvement over the null becomes statistically significant). Thus, any conclusion made from a test in this context is dubious, since the asymptotic p-value will always go to 0. Therefore, we used an approach based on confidence intervals of the overall dose-response curve, which do not have this particular pathology. Several genes of interest were chosen for examination based on their association with leukemogenesis or benzene metabolism. The observed significant decrease in CEBPA expression at benzene levels of around 0.1 ppm may be important in light of the fact that reduced CEBPA gene expression has been associated with increased risk of leukemia [55]. Changes in the expression of CYP2E1 were observed at levels of air benzene concentration as low as 0.01 ppm. As benzene metabolism occurs principally in the liver [62] and also in the lung [63], [64], with secondary metabolism occurring in the bone marrow [65], [66], [67], the implication of altered expression of CYP2E1 in peripheral blood is not entirely clear.

In order to permit phenotypic anchoring of the observed dose-dependent changes in gene expression, the responses of counts of B-cells, white blood cells and the ratio of the counts of CD4 cells to CD8 cells, are estimated using the Bootstrap-SuperLearner approach (data not shown). The observed decreases in these cell counts have been previously reported [7]. Mean changes in gene expression can thus be associated with corresponding changes in mean cell counts. For example, a 0.75 expected fold change of CEBPA gene expression at 1 ppm of air benzene would be associated with a mean decrease of 600 white blood cells/µl or a decrease of 0.2 in the ratio of CD4 to CD8 cells.

In summary, this work presents a new approach, which is the combination of the choice of a statistical parameter to be estimated, the methods used to estimate the data generating distribution (our parameter is only a targeted part of that distribution), and rigorous and robust methods for deriving inference, applied to the scientific question of estimating the dose-dependent biological responses resulting from exposure to benzene in the air. This work extends our previous analyses of benzene-induced differential gene expression in occupationally exposed workers and demonstrates that the differential expression of the majority of genes is independent of changes in cell counts of various blood cell types; that many differentially expressed genes and disease-relevant pathways display an apparently supra-linear response; and, that benzene alters these pathways and genes at exposure levels as low as 0.1 ppm. However, limitations in the statistical models and in the interpretation of some of these findings suggest that studies with a larger number of samples from individuals exposed to benzene in the low-dose range between 0.01 and 1 ppm are needed to clarify and confirm our interpretations. More precise exposure measurement and/or estimation in the low-dose region are needed to clarify the nature of the dose-response relationship of gene alteration in the low-dose range.

## Supporting Information

Figure S1 **Distribution of intra-class coefficients of the chip, subject, labeling and hybridization random effects.**
(PDF)

Figure S2
**Pathways-Principal Components-based Response.** Non-parametric model fits to the marginal association of the expression of the probes corresponding to the genes involved in the a. B-cell receptor signaling, b.Toll-like receptor signaling, c. Steroid Hormone bio-synthesis and d.Maturity onset of diabetes pathways with air-benzene concentrations in parts per million. The continuous fits in the two subplots use the first and second eigenvectors (that are slightly modified, see Material and Methods section) respectively from the eigenvector matrix, 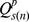 given in Equation (5). The elements of the individual eigenvectors are treated as the pathway response at the corresponding dose. The subscript *p* corresponds to the pathway under consideration and superscript *s* to a given bootstrap sample. The bold red line correspond the mean parameter estimates across the bootstrap samples and the bold black lines represent the corresponding 95% confidence intervals for the mean parameter estimates. The small vertical ticks on the x-axis denote doses to which one or more subjects in the study were exposed and consequently the doses for which data for all covariates under consideration were available. The three red ‘x’s above these ticks indicate the doses that there used to compare the rate of change of the marginal effect of benzene exposure from 0.001 to 1 ppm air benzene to the corresponding rate from 1 to 10 ppm air benzene.(PDF)

Figure S3
**Pathways-Clusters of probes/genes.** Non-parametric model fits to the marginal association of the expression of the probes corresponding to the genes involved in the a. B-cell receptor signaling, b.Toll-like receptor signaling, c. Steroid Hormone bio-synthesis and d.Maturity onset of diabetes pathways with air-benzene concentrations in parts per million. The probes are clustered based on the distance between the corresponding rows of the matrix, 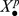 given in Equation (6). The figure is a visual representation of the distance matrix between all the probes/genes in the pathway. The color of the *(i,j)^th^* position of the distance matrix is a measure of how close probes *i* and *j* are to each other based on their response across the dose range. The color ranges from white to red. The closer the pair of probes is two each other, the greater the intensity of red at the corresponding position. The dashed black lines correspond to boundaries of clusters of probes as determined by the HOPACH algorithm [47].(PDF)

Table S1 **List of supervised learning algorithms.**
(XLSX)

Table S2 **Fixed effects estimates for the mixed model in** **Equation (1** **).**
(XLSX)

Table S3 **p-Values for KEGG pathways.** The p-values were computed using the *SEPEA_NT3* procedure [26] based on results of differential from expression (in at least one of the four benzene exposure groups) from the linear mixed models with (L1) and without (L0) using the blood cell counts as potential confounders of gene expression. Also listed are the p-values obtained the KEGG pathway enrichment using genes commonly identified by both models, unique to the model (L0) and unique to the model (L1).
(XLSX)

Table S4
**Median and 95% confidence interval (CI) estimates of the rate of change of marginal effect of benzene exposure below 1 ppm (** 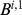 **/** 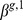 **– see** **equations (10** **) and (13)) and above 1** **ppm (** 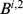 **/** 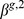 **- see** **equations (11** **) and (14)) and the change in absolute rate of change of the marginal effects from below 1** **ppm to above 1** **ppm (** 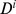 **/** 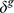 **– see** **equations (12** **) and (15)) for the first two principal components of the for the B-cell receptor signaling, Toll-like receptor signaling, Steroid hormone synthesis and Maturity onset of diabetes pathways.**
(XLSX)
