# Supplementary figures and images for: Characterization of Changes in Gene Expression and Biochemical Pathways at Low Levels of Benzene Exposure
Source: PLoS One. 2014 May 1;9(5):e91828. doi: 10.1371/journal.pone.0091828 (PMC4006721; doi:10.1371/journal.pone.0091828)

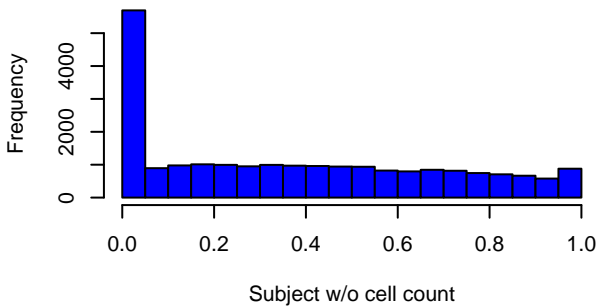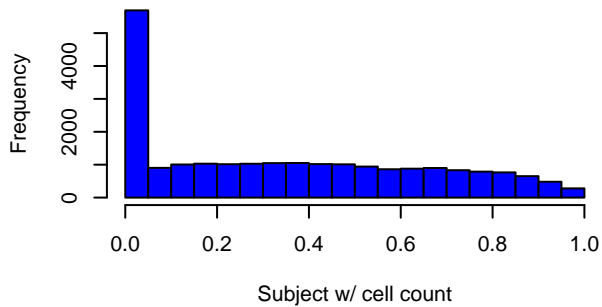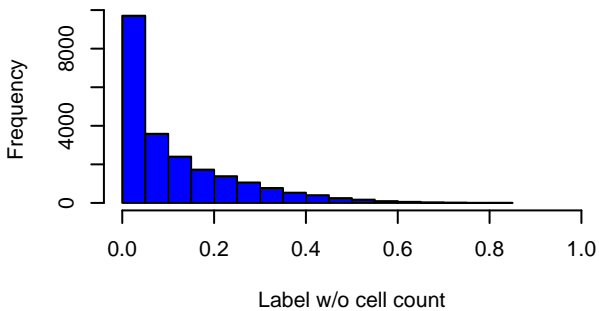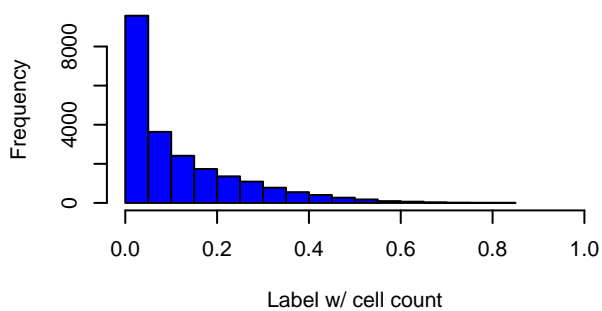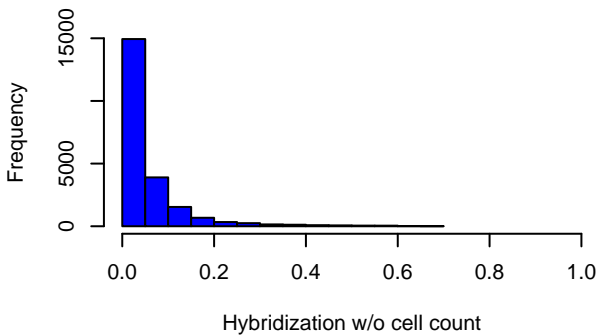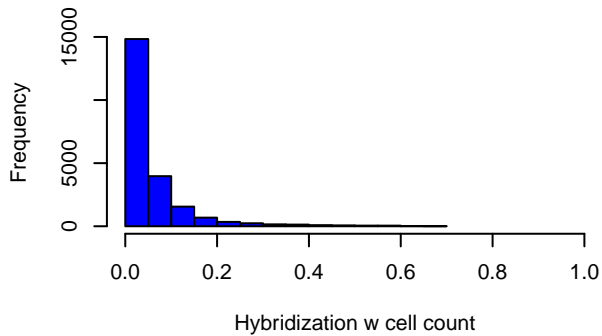

Supplement: Figure S1 — Distribution of intra-class coefficients of the chip, subject, labeling and hybridization random effects. (PDF) [file pone.0091828.s001.pdf]
