# Supplementary material for: Characterization of Changes in Gene Expression and Biochemical Pathways at Low Levels of Benzene Exposure
Source: PLoS One. 2014 May 1;9(5):e91828. doi: 10.1371/journal.pone.0091828 (PMC4006721; doi:10.1371/journal.pone.0091828)

Figure S2(a-d): Pathways- Principal Components-based response

a. B-cell receptor signaling pathway

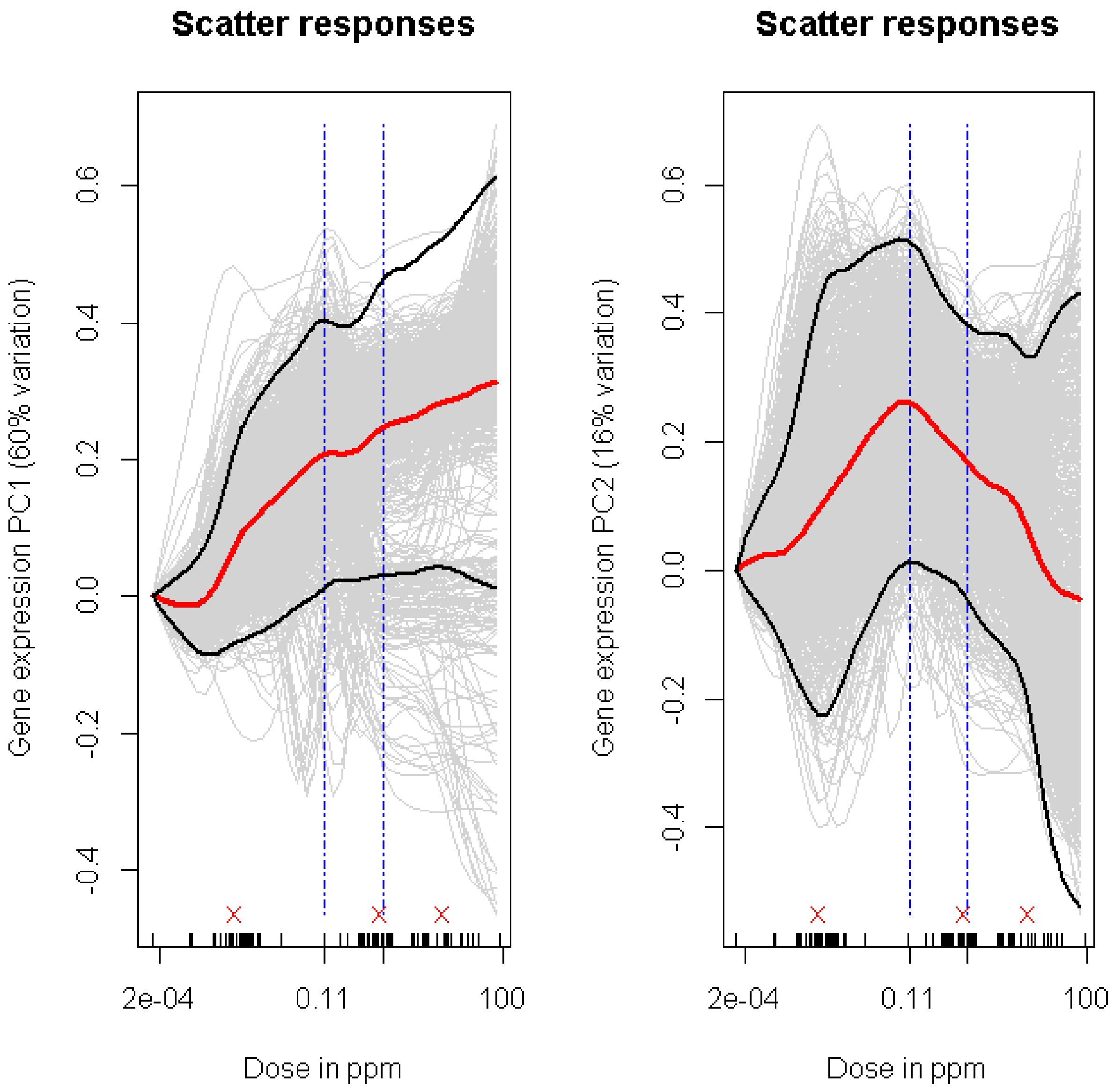

b. Toll-like receptor signaling pathway

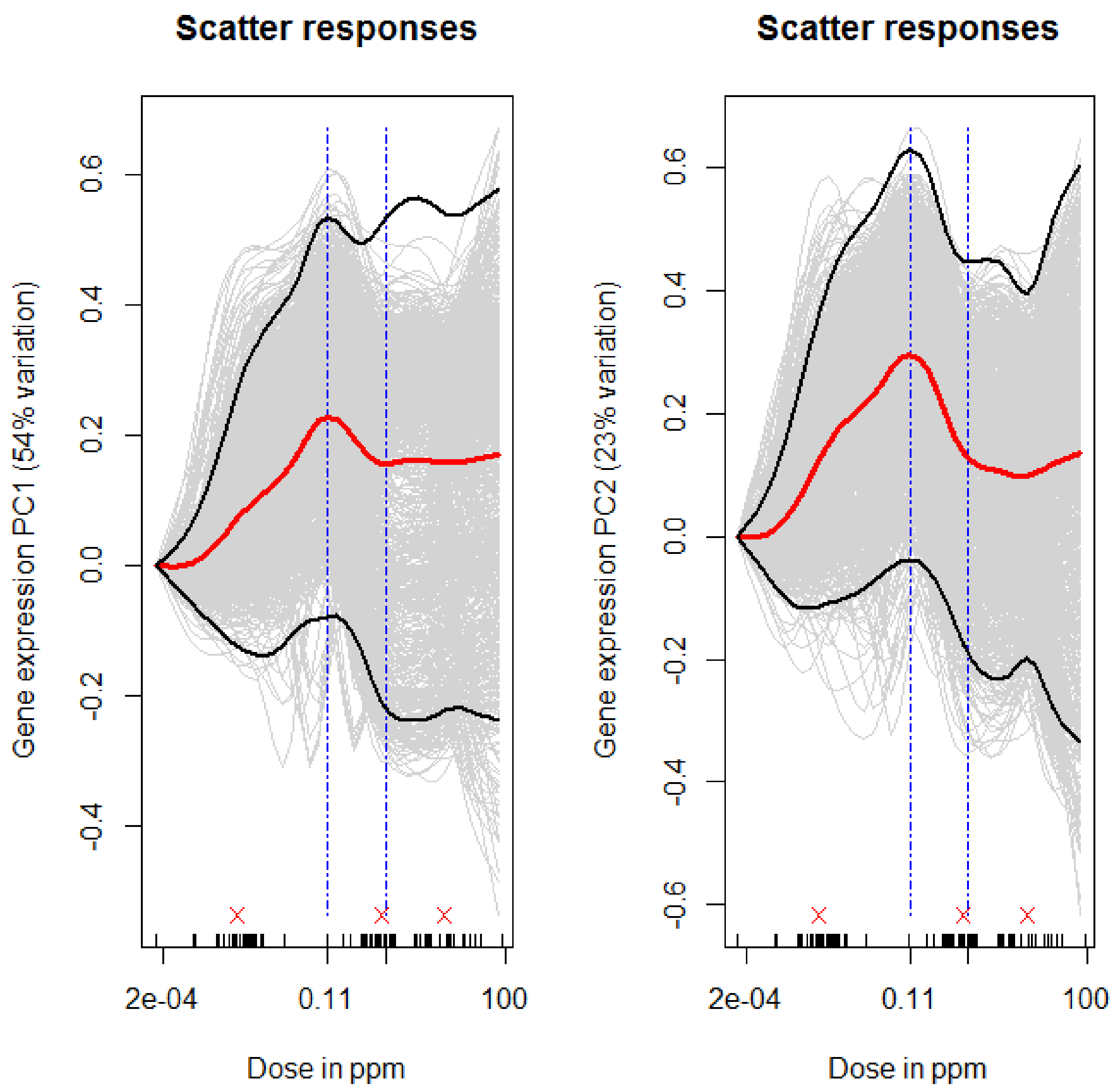

c. Steroid hormone biosynthesis pathway

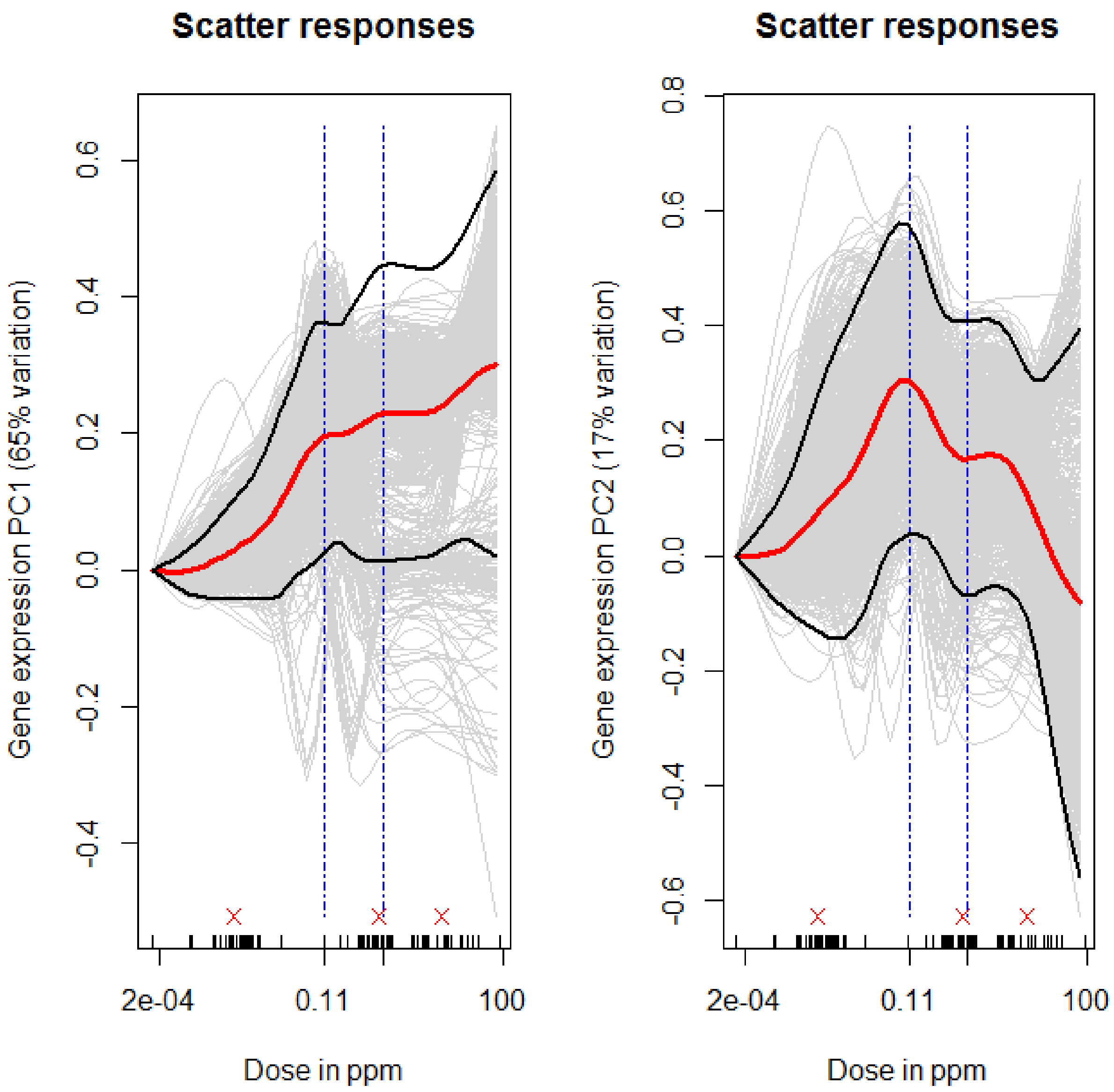

d. Maturity onset of diabetes pathway

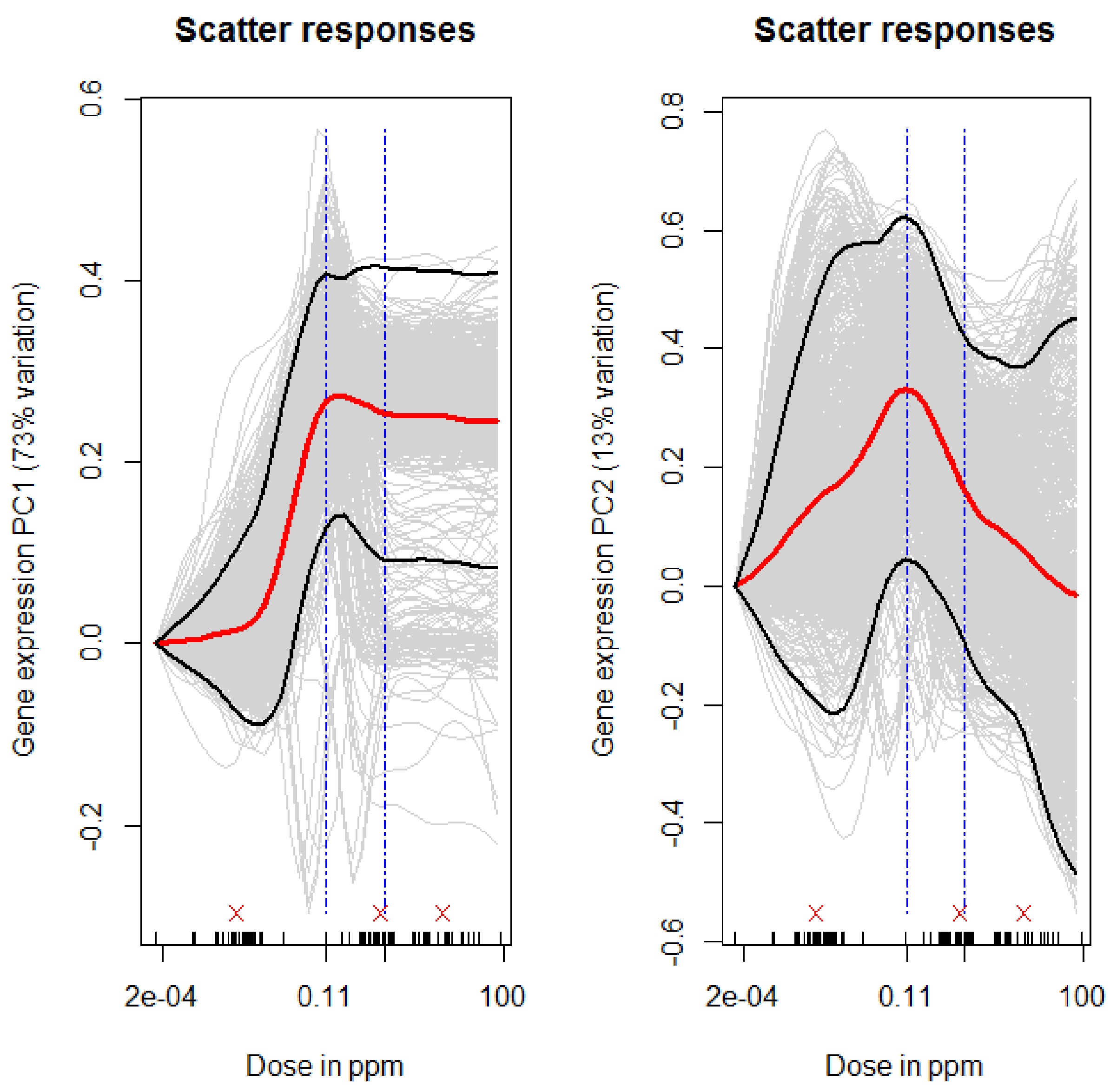

Supplement: Figure S2 — Pathways-Principal Components-based Response. Non-parametric model fits to the marginal association of the expression of the probes corresponding to the genes involved in the a. B-cell receptor signaling, b.Toll-like receptor signaling, c. Steroid Hormone bio-synthesis and d.Maturity onset of diabetes pathways with air-benzene concentrations in parts per million. The continuous fits in the two subplots use the first and second eigenvectors (that are slightly modified, see Material and Methods section) respectively from the eigenvector matrix, given in Equation (5). The elements of the individual eigenvectors are treated as the pathway response at the corresponding dose. The subscript p corresponds to the pathway under consideration and superscript s to a given bootstrap sample. The bold red line correspond the mean parameter estimates across the bootstrap samples and the bold black lines represent the corresponding 95% confidence intervals for the mean parameter estimates. The small vertical ticks on the x-axis denote doses to which one or more subjects in the study were exposed and consequently the doses for which data for all covariates under consideration were available. The three red ‘x’s above these ticks indicate the doses that there used to compare the rate of change of the marginal effect of benzene exposure from 0.001 to 1 ppm air benzene to the corresponding rate from 1 to 10 ppm air benzene. (PDF) [file pone.0091828.s002.pdf]
