# Supplementary material for: Characterization of Changes in Gene Expression and Biochemical Pathways at Low Levels of Benzene Exposure
Source: PLoS One. 2014 May 1;9(5):e91828. doi: 10.1371/journal.pone.0091828 (PMC4006721; doi:10.1371/journal.pone.0091828)
Supplement: Figure S3 — Pathways-Clusters of probes/genes. Non-parametric model fits to the marginal association of the expression of the probes corresponding to the genes involved in the a. B-cell receptor signaling, b.Toll-like receptor signaling, c. Steroid Hormone bio-synthesis and d.Maturity onset of diabetes pathways with air-benzene concentrations in parts per million. The probes are clustered based on the distance between the corresponding rows of the matrix, given in Equation (6). The figure is a visual representation of the distance matrix between all the probes/genes in the pathway. The color of the (i,j)th position of the distance matrix is a measure of how close probes i and j are to each other based on their response across the dose range. The color ranges from white to red. The closer the pair of probes is two each other, the greater the intensity of red at the corresponding position. The dashed black lines correspond to boundaries of clusters of probes as determined by the HOPACH algorithm [47]. (PDF) [file pone.0091828.s003.pdf]

Figure S3(a-d): Pathways-Clusters of probes/genes

a. B-cell receptor signaling pathway

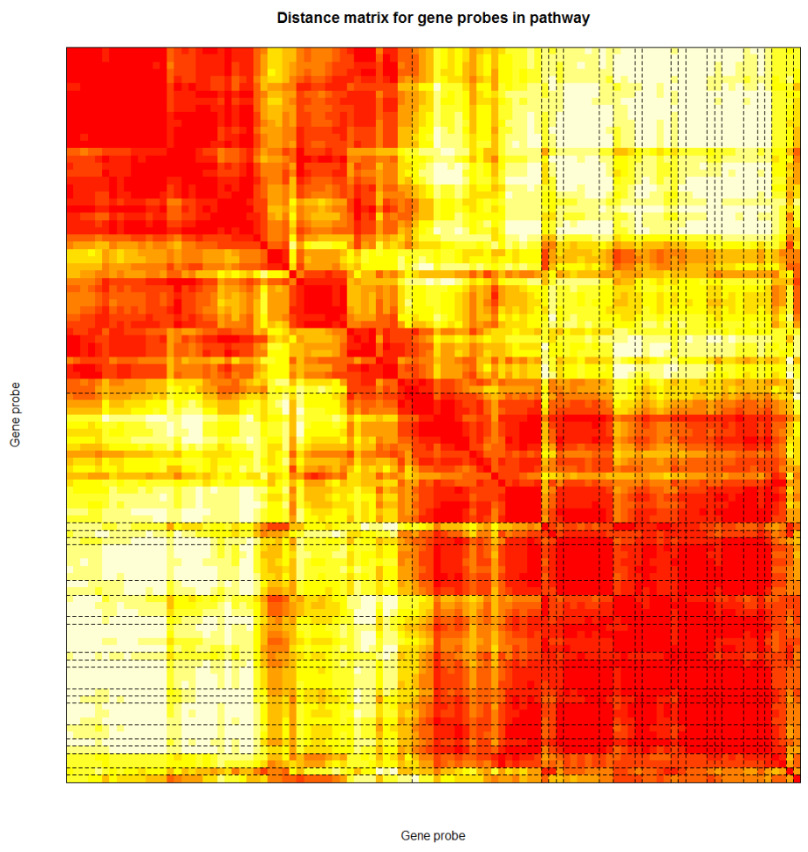

b. Toll-like receptor signaling pathway

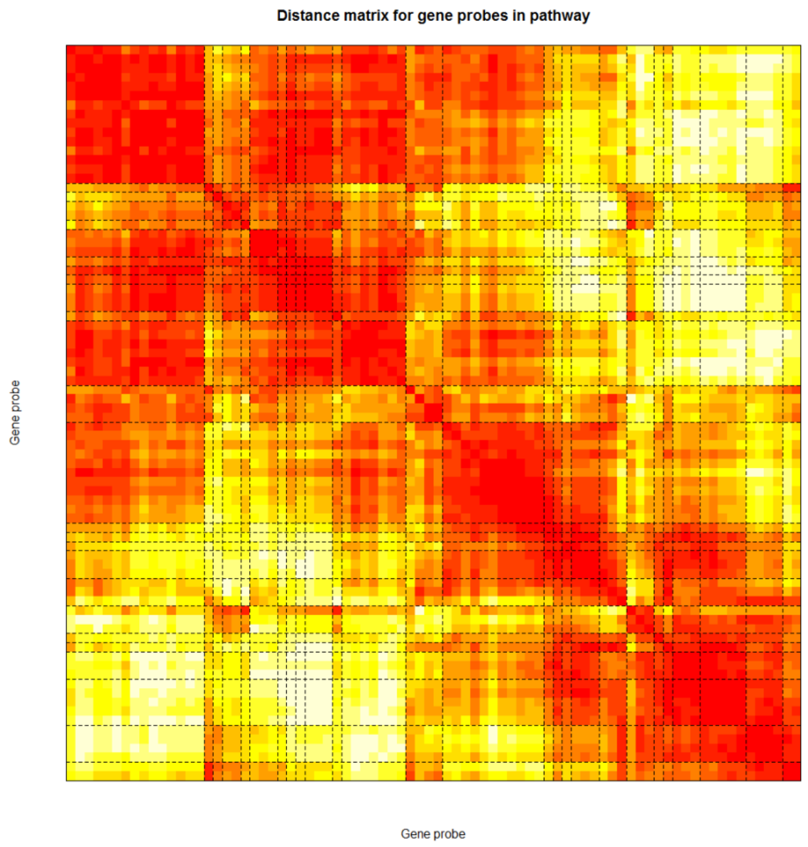

c. Steroid hormone biosynthesis pathway

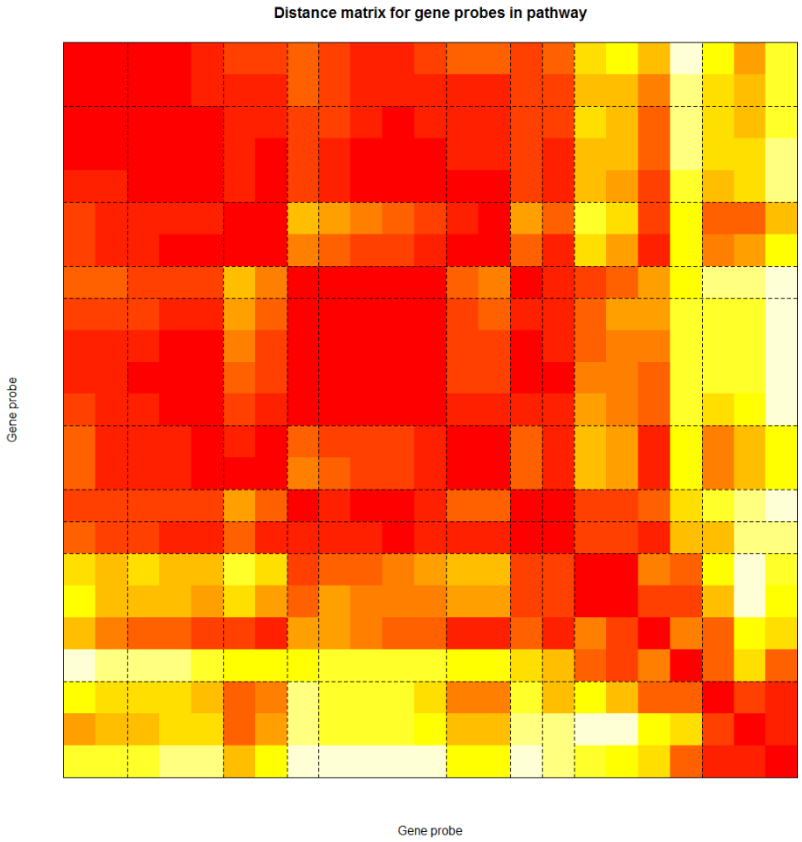

d. Maturity onset of diabetes pathway

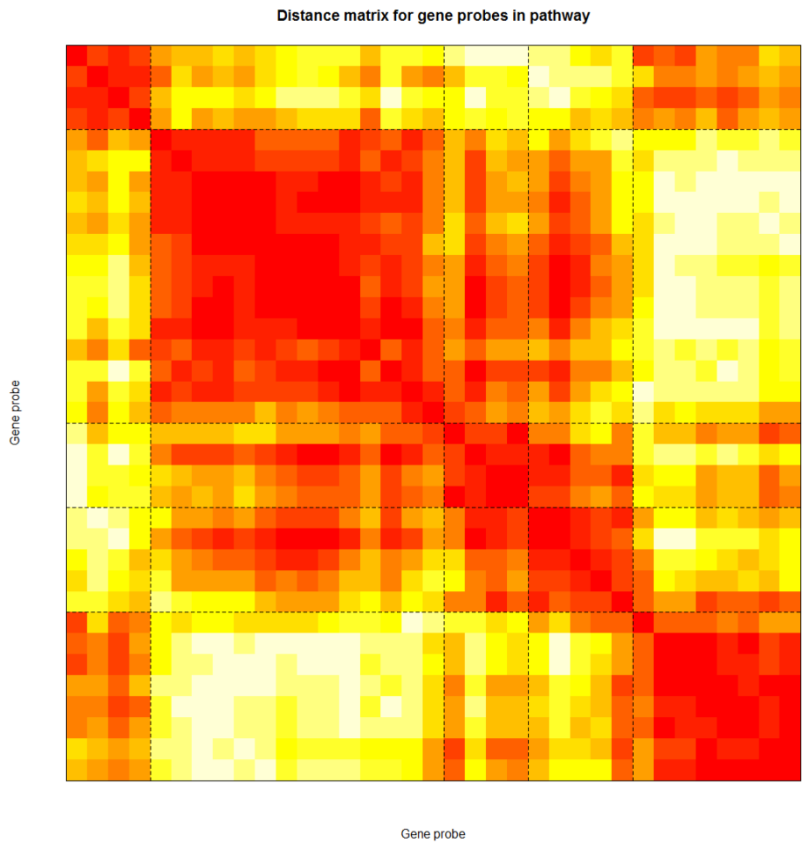
Distance matrix for gene probes in pathway
Gene probe
Gene probe
